# Reference genes for normalization of gene expression studies in human osteoarthritic articular cartilage

**DOI:** 10.1186/1471-2199-9-17

**Published:** 2008-01-29

**Authors:** Manuel Pombo-Suarez, Manuel Calaza, Juan J Gomez-Reino, Antonio Gonzalez

**Affiliations:** 1 Laboratorio de Investigacion 2 and Rheumatology Unit, Hospital Clinico Universitario de Santiago, Santiago de Compostela, Spain; 2 Department of Medicine, University of Santiago de Compostela, Spain

## Abstract

**Background:**

Assessment of gene expression is an important component of osteoarthritis (OA) research, greatly improved by the development of quantitative real-time PCR (qPCR). This technique requires normalization for precise results, yet no suitable reference genes have been identified in human articular cartilage. We have examined ten well-known reference genes to determine the most adequate for this application.

**Results:**

Analyses of expression stability in cartilage from 10 patients with hip OA, 8 patients with knee OA and 10 controls without OA were done with classical statistical tests and the software programs geNorm and NormFinder. Results from the three methods of analysis were broadly concordant. Some of the commonly used reference genes, GAPDH, ACTB and 18S RNA, performed poorly in our analysis. In contrast, the rarely used TBP, RPL13A and B2M genes were the best. It was necessary to use together several of these three genes to obtain the best results. The specific combination depended, to some extent, on the type of samples being compared.

**Conclusion:**

Our results provide a satisfactory set of previously unused reference genes for qPCR in hip and knee OA This confirms the need to evaluate the suitability of reference genes in every tissue and experimental situation before starting the quantitative assessment of gene expression by qPCR.

## Background

Osteoarthritis (OA) is the most common rheumatic disease and a leading cause of disability in the elderly [1]. It involves ligaments, subchondral bone, synovium and cartilage [2,3]. Most research in OA has been focused in articular cartilage where the disease becomes highly evident in its late stages. Biochemical changes in chondrocytes and extracellular matrix components are followed by macroscopic lesions including thinning, fibrillation, fissuring and erosion of cartilage that will eventually lead to denudation of subchondral bone. These changes result from active processes that involve matrix destruction and inefficient repair [4-6]. Progress in the management of OA requires better knowledge of the regulation of these processes as they could have a different impact depending on its etiology. The commonest form of OA is idiopathic and appears only in the elderly. Nevertheless, some forms of OA have a genetic cause or are secondary to rheumatic, endocrine, metabolic or neuropathic diseases or to local factors like trauma, infection or avascular necrosis [7]. This variety of etiologies as well as OA chronic evolution, its heterogeneity in different joints, and the possibility of wide differences in gene expression between different disease stages or areas of cartilage complicate OA research [8-10]. There are not generally accepted methods to address these issues.

In recent years, it has become possible to study satisfactorily gene expression in cartilage. A major problem has been the difficulty in obtaining RNA due to the unique characteristics of human cartilage as low cell content, collagenous matrix and richness in proteoglycans that co-purify with RNA [11]. Methods improving RNA yield and quality as well as methods of cDNA amplification by in vitro transcription that can compensate for the poor content of RNA in human cartilage have been reported [11-14]. Techniques allowing precise quantification of gene expression are also available, microarrays for a large number of genes or quantitative real-time PCR (qPCR) for individual genes [5,9,10,15-18] This latter has a main role in studies focused on a few genes and to validate results from microarray studies. However, the full potential of qPCR cannot be obtained if specific care is not taken [19,20].

The absolute amount of a gene RNA can be quantified by qPCR, but this is seldom done because between-sample variation in RNA extraction, reverse transcription and PCR efficiency make the procedure inaccurate. Also, requirement of gene-specific calibration curves makes it too complex for many laboratories [20,21]. The commonest alternative is relative quantification, in which normalization by endogenous reference genes allows comparison between samples but not between genes. In this approach, selection of the reference gene is critical because its expression should be invariable under the conditions of study [22-25]. The suitability of reference genes in cartilage has not been addressed previously, though a study on chondrocytes has recently been published [26], and this lack of analysis is not without risks. Most reference genes in common use were selected because they are housekeeping genes, that is, they are widely and constitutively expressed in many tissues and stages of development. However, these genes are also regulated [21]. They were useful for Northern blot and RNase protection assays but not for the more sensitive real-time qPCR. Small changes in expression of the reference genes will lead to wrong conclusions, as has been shown in many areas of research [25,27-29]. Therefore, expression stability of the prospective reference genes should be explored in each specific tissue and type of experiment [22,30,31]. In fact, most authors that have investigated this area agree in the need of using more than a single reference gene to obtain high quality data [30,32]. In our study, we have explored ten well-known reference genes to identify the most suitable for normalization of qPCR data from human cartilage obtained from the hips and knees of elderly healthy subjects and OA patients.

## Results

The prospective reference genes included in this study covered a wide range of expression levels in articular cartilage (mean Ct values ranging from 18 to 36). Results from individual samples showed a uniform dispersion around the mean without any marked skeewing (not shown). qPCR replicates showed very low variability, with a mean coefficient of variation (CV) of 1.08% ± 1.2 (standard deviation, SD). When the raw individual values were stratified by group of samples-hip OA, knee OA, or hip controls – there were differences in the HPRT1 and 18S RNA values. After data transformation, which involved correction by well-specific efficiency and determination of the relative value of each sample in relation to the gene-specific median of all samples, the Mann-Whitney U test showed that 18S RNA was expressed at significantly lower levels in hip and knee OA cartilage than in control hip cartilage (p = 0.03). This difference indicated a possible source of spurious results if 18S RNA is used for normalization in qPCR across the mentioned groups of samples.

**Figure 1:**
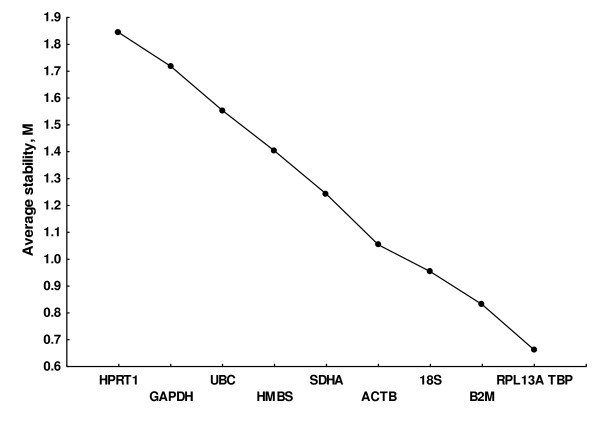
**Average expression stability, M, of prospective reference genes**. Values of M were obtained with the geNorm software that compares gene expression without accounting for experimental groups and proceeds to the stepwise exclusion of the genes whose relative expression levels are more variable among tissue samples. Lower values of M correspond to the most stable genes, hence the most suitable for normalization.

**Figure 2:**
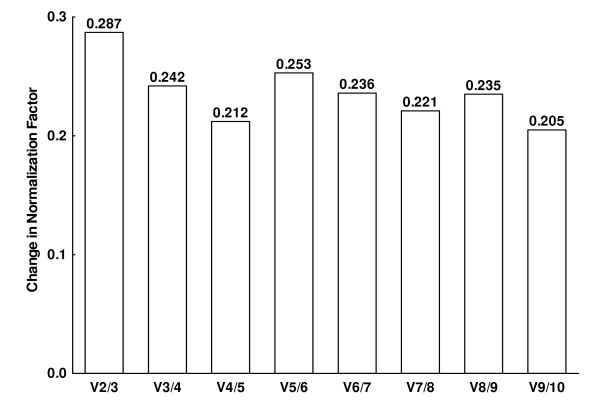
**Evaluation of the optimum number of reference genes according to the geNorm software**. The magnitude of the change in the normalization factor after the inclussion of an additional reference gene reflects the improvement obtained. V*i*/*i+1 *represent the models being compared: those with *i *and *i+1 *reference genes.

Transformed expression data were further analyzed with the geNorm software that determines a gene expression stability measure (M) for each candidate reference gene based on the average pairwise variation between a particular gene and all other control genes [30]. For this analysis, results from the 28 samples were considered together. As shown in Figure 1, the least stable gene with this approach was HPRT1 and the most stable genes were RPL13A and TBP. All the others were between them without discernible groups among them. The same software provides an estimation of the optimum number of reference genes. This estimation is obtained by analysis of the changes in the normalization factors obtained by adding successively the next most stable gene of the set. A large change after a step indicates that the added gene has a significant effect and should be included in the normalization factor. Changes in our data set were rather uniform (Figure 2) with a possible optimum point after including the four most stable genes: TBP, RPL13A, B2M and 18S RNA.

We were concerned by the possibility that sex differences between control and OA samples could affect the choice of most stable genes. However, separate geNorm analyses in men and women provided the same set of three most stable genes, TBP, RPL13A and B2M. The geNorm analysis was also repeated after excluding the HPRT1 and 18S RNA genes given the mentioned differences in expression between groups of samples. In this new analysis, the most stable genes were RPL13A and TBP, followed by B2M, concordant with the previously found.

**Figure 3:**
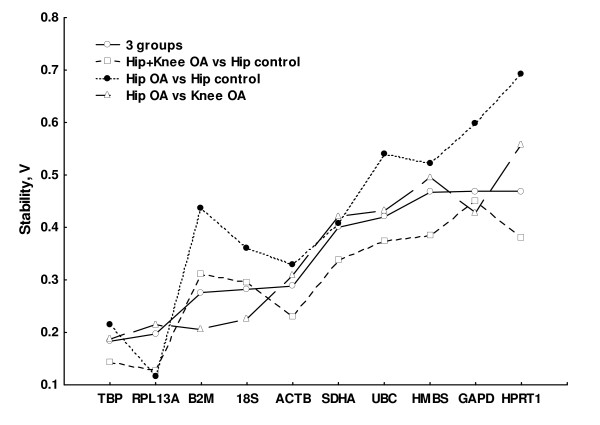
**Stability of the prospective reference genes depending on the way the samples were grouped**. The stability values, V, were obtained with NormFinder that combines intra- and intergroup variation in the expression of each gene. Results from all the meaningful comparisons of samples from the Hip OA, Knee OA and Hip Control groups are presented. Lower values of V correspond to the most stable genes, hence the most appropriate for normalization.

Given the distribution of our samples in three clinically different groups, it was interesting to analyse qPCR data with the NormFinder program that provides a stability value considering sample stratification in groups [32]. The best two-gene combination considering the three groups of samples (hip OA, hip controls and knee OA), was TBP and RPL13A, corresponding to the geNorm result. A general concordance was also found when considering the results of each gene individually (Figure 3). There were the same less stable genes including GAPDH, HPRT1, HMBS, UBC and SDHA and the same most stable genes including TBP, RPL13A, B2M, 18S RNA and ACTB. We have also explored how the stability values changed if the samples were considered in groups that could be clinically relevant: hip OA vs hip control samples, hip OA and knee OA together vs hip controls, and hip OA vs knee OA. In all these comparisons, the genes with the most stable expression were RPL13A, TBP, B2M, ACTB and 18S RNA, with their order changing slightly in function of the groups considered (Figure 3).

## Discussion

A first consideration in the analysis of our results is that the prospective reference genes that we have analyzed had already been selected in previous studies because of their utility for this function [25,28,30,32], they have relative stable expression and we did not expect large differences between them. Also, these genes are from different functional families and not known to be coregulated. This implies that each one provides independent and complementary information, which is an important requisite for geNorm analysis. A second important consideration is that we have made efforts to minimize every known source of experimental variation by DNAse digestion, adjusting the amount of input RNA, using two-step reverse transcription polymerase chain reaction (RT-PCR) and by correcting raw results for PCR efficiency [19-21,24,33]. These steps are a requisite for the assumption that observed results reflect true gene expression. We found specially necessary to include a DNAse digestion step because human genome has many processed pseudogenes inserted by retrotransposition that are amplified in PCR even with intron-spanning primers [30].

The systematic selection of the best reference genes for real-time qPCR has been approached with different methods. All of them look for stability of the expression levels, by either absence of differences between clinically relevant groups [28], or relative stability in relation to other reference genes [30,34] or in relation to clinically relevant groups [32]. These analyses have been facilitated by the free availability of programs and by the description of their principles and use [30,32,34]. There are no definitive reasons to prefer one method over the others as their relative strengths depend on the circumstances and we have used three of the best-grounded: a conventional statistical test to compare clinically relevant groups and the geNorm and NormFinder softwares. Results from the three were broadly similar, though the conventional statistical analysis lacked sensitivity. The independence of our results from the analysis method gives credence to the conclusions.

The most striking result was the poor performance of some commonly used reference genes. A special case was GAPDH that is widely used in many areas of research [22] and is one of the best reference genes in many tissues [30]. Nevertheless, there have been also previous examples of this gene leading to wrong results due to its lack of stability in specific experimental conditions [22]. In our study, GAPDH was not among the best reference genes in any of the analyses done. Other two commonly used reference genes, ACTB and 18S RNA [22], performed better in our tests but they were not among the more stable genes in most comparisons. These results confirm, once more, the need to evaluate the reference genes in each experimental setting. A particularly striking example in this regard, is the contrast between our results and the reported in prostate cancer tissue, where HPRT1 was the most stable gene, and RPL13A and ACTB were the most unstable [28]. In our experiments, their ranks were reversed, i.e. HPRT1 was the most unstable and RPL13A one of the most stable genes.

Best reference genes in articular cartilage from elderly subjects were among the less commonly used: TBP, RPL13A and B2M. Best results will be obtained by combining two or three reference genes as emphasized by several authors [30,32]. We propose that for general studies of cartilage from elderly subjects a combination of TBP and RPL13A could be a good starting point, with the inclusion of B2M if practical. For specific comparisons other combinations could be more appropriate.

Finally, it is necessary to take into account some limitations of our study. First, we have included a limited array of prospective reference genes. Other genes have been proposed for use in qPCR, and it is possible that some of them are better candidates for articular cartilage studies in elderly subjects. Microarray data from cartilage, that now start to be published [5,10] will provide clues for the identification of the best candidates. Second, our results only apply directly to articular cartilage with a focus in OA of large joints. In particular, collection of samples from surgical procedures dictated that all donors were older than 60 years and that OA samples were of an advanced disease stage. This mimics most of the studies in cartilage in OA. However, it is unclear how well our results could be extended to other joint areas, patients with different ages or OA at early stages. Nevertheless, our study can serve as a guide for any kind of cartilage study, and reference genes could be used once tested for low M values. It is also unclear to what extent results obtained with SYBR Green quantification will be applicable to other relative qPCR techniques.

## Conclusion

Precise assessment of gene expression in cartilage samples from elderly subjects requires selection of suitable reference genes. Some of the commonly used performed poorly, questioning the accuracy of previous reports. Combinations of the previously unused genes TBP, RPL13A and B2M were found to perform addequately and are recommended to improve evaluation of gene expression in OA research. In studies involving only the hip joint, TBP and RPL13A are the best choice.

## Methods

### Cartilage samples

Human articular cartilage was obtained from femoral heads and from the femoral condyles and tibial plateaux of patients undergoing total joint replacement surgery at the Hospital Provincial de Conxo (Santiago de Compostela, Spain). Causes of joint replacement were primary OA or fracture of the femoral neck. All subjects have been already diagnosed by the clinicians following them from anamnesis, and clinical and radiographic data. One of us, a licensed rheumatologist, reviewed all this previous information including the radiographs previous to joint surgery. He also conducted a new anamnesis of each subject following a specific questionnaire and performed a new clinical evaluation when possible (not yet operated). Confirmation of diagnosis was made at the time of joint removal based on macroscopic findings. No histology examination was done. The fracture patients were selected to exclude history of joint disease, and radiographic and macroscopic signs of joint lesions. No patients with unclear classification as either OA or non-OA were included. After selection with these criteria, 10 hip OA, 8 knee OA and 10 hip fracture samples, age- and sex-adjusted, were used. No knee control samples were available for study. Main characteristics of the cartilage donors are shown in Table 1. This study received the approval of the Ethical Committee for Clinical Research of Galicia and all participants gave their written informed consent. All participants were of Spanish ancestry.

**Table 1:** Characteristics of the cartilage donors included in the study

	Group		
	
Parameter	Knee OA	Hip OA	Hip control
No. of patients	8	10	10
Age, median (range) years	72 (67–77)	75 (66–85)	81 (72–91)
No. male/female	5/3	5/5	3/7
Collins grade, average (range)	3.4 (2–4)	3.7 (2–4)	0.7 (0–1)

### Cartilage dissection and evaluation

Intact femoral heads and knees were washed and kept in sterile PBS at 4°C. Surface of the cartilage was carefully examined and graded by the macroscopic visual Collins' scale modified by Muehleman [35]. Briefly, grade 0: no signs of cartilage lesions; grade 1: very limited disruptions of the articular surface with no changes in surface geometry; grade 2: deep fibrillation and fissuring, early marginal hyperplasia and possibly, small osteophytes; grade 3: extensive fibrillation and fissuring, 30% or less of the articular cartilage surface eroded down to the subchondral bone, and osteophytes; and grade 4: lips or shelves at the articular margin, greater than 30% of the articular surface eroded down to the subchondral bone and gross geometric changes and osteophytes. Cartilages with Collins grades 0 and 1 are considered normal, while cartilages of grade 2 and higher are considered degenerated. Given the advanced stage of disease in the OA samples there were areas of the joint surface without cartilage. All the remaining cartilage was removed from the bone using a scalpel, chopped into 2–5 mm pieces and snap-frozen in liquid nitrogen within 6 hours of surgery. For consistency, we took also all available cartilage from control donors. Special care was taken to exclude fibrotic tissue or any subchondral bone contamination. Tissue pieces were stored at -80°C until further processing.

### RNA extractions

RNA extractions from articular cartilage were performed following the method of Price et al [12] with the addition of a DNase digestion step. Frozen cartilage was weighed and 1 g was ground using a stainless-steel mortar and pestle that were liquid nitrogen-cooled. After initial extraction in TRI Reagent (Sigma, Saint Louis, MO), the aqueous phase was mixed with a half volume of 100% ethanol and further purified on silica-gel-based membranes using the RNeasy Plant Mini Kit (Qiagen, Valencia, CA) according to the manufacturer's instructions. A DNase I (Qiagen, Valencia, CA) digestion step was performed on the spin column. Concentration of the isolated RNA and the 260/280 nm absorbance ratio were measured with the NanoDrop^® ^ND-1000 spectrophotometer (NanoDrop Technologies, Wilmington, DE, USA). Samples with 260/280 ratio < 1.90 were discarded.

### RT-QPCR

RNA was reverse transcribed to cDNA using Transcriptor Reverse Transcriptase (Roche Applied Science, Barcelone, Spain) and random hexamers in a total volume of 20 μl according to the manufacturer's instructions. Limited RNA quantities dictated input RNA amounts to be 220 ng. Complementary DNA was stored at -20°C until 0.5 μl were used in each downstream PCR. Oligonuleotide primers were designed using Primer3 software [36] in different exons to help prevent amplification of genomic DNA. PCRs were performed using DyNAmo™ SYBR^® ^Green qPCR kit (Finnzymes, Espoo, Finland) and Chromo4™ real-time PCR detection system (MJ Research, Basel, Switzerland). The PCR reaction volume was 10 μl with 0.1 μM of the primers. Cycle conditions were set as an initial denaturation step for 10 min at 95°C, followed by 40 cycles of 10 s at 94°C for template denaturation, 15 s for annealing and 10 s at 72°C for extension. Table 2 shows the annealing temperatures and the MgCl_2 _concentrations specific for each set of primers. All reactions were run in duplicate and all samples were analyzed in the same run to exclude between-run variations. Each RNA sample was controlled for genomic DNA contamination for each gene-specific PCR by a reaction well without reverse transcription. Reagent contamination was also examined by a reaction mix without template. Specificity of the PCR reactions was confirmed by melting curve analysis of the products as well as by size verification by DNA electrophoresis in agarose gels.

**Table 2:** Real-time quantitative PCR characteristics

Gene	Accesion No.	Forward primer	Reverse primer	Annealing °C	MgCl_2 _mM	Amplicon size (bp)
HMBS	NM_000190	ggcaatgcggctgcaa	gggtacccacgcgaatcac	56	2.5	64
TBP	NM_003194	tgcacaggagccaagagtgaa	cacatcacagctccccacca	56	2.5	132
B2M	NM_004048	atgagtatgcctgccgtgtga	ggcatcttcaaacctccatg	56	2.5	101
ACTB	NM_001101	ggcatcctcaccctgaagta	ggggtgttgaaggtctcaaa	56	2.5	203
HPRT1	NM_000194	tgctcgagatgtgatgaagg	tcccctgttgactggtcatt	56	2.5	192
RPL13A	NM_012423	aaaaagcggatggtggttc	cttccggtagtggatcttgg	56	4.5	168
SDHA	NM_004168	tggacctggttgtctttggt	agtcgcagttccgatgttct	56	3.5	166
UBC	NM_021009	atcgctgtgatcgtcacttg	tccagcaaagatcagcctct	64	3.5	164
18S	NM_022551	atccctgaaaagttccagca	ccctcttggtgaggtcaatg	56	2.5	186
GAPDH	NM_002046	gagccacatcgctcagacac	catgtagttgaggtcaatgaagg	60	3.5	150

### PCR efficiency and Cycle threshold (Ct) determination

PCR efficiency was calculated with the LinRegPCR program [33] from raw fluorescence data taken from the Chromo4™ real-time PCR detection system. According with this method PCR efficiency is the slope of the straight line that best fit the log-linear part of the amplification curve. Mean efficiencies were determined in sample duplicates and used to adjust Ct values. Ct values, the cycle number at which the fluorescence signal of the sample exceeds background fluorescence, were used for the quantitative comparison of the amplification rates. They were obtained using Opticon Monitor™ version 3.0 software, provided with the Chromo4™ real-time PCR detection system. After baseline subtraction, threshold lines were manually established for each gene to cross the the log-linear part of the fluorescence curves. Mean Ct values of the duplicates were determined and transformed into relative quantities.

### Data analysis

Mann-Whitney U tests were performed with Statistica, version 7 (Statsoft, Tulsa OK). The softwares geNorm™, version 3.4 [30] and NormFinder [32] were used to calculate stability of the candidate reference genes. The first, geNorm, relies on the principle that the expression ratio of two reference genes should be identical in all samples, regardless of the experimental condition. It calculates the expression stability measure (M) for the set of candidate reference genes and by stepwise exclusion of the least stable gene in each step arrives to the the most stable pair of reference genes. It provides also a way to estimate the best number of required reference genes. NormFinder follows a different approach: it calculates a stability value for each individual candidate reference gene taking into account separation of samples in the different groups that are of interest in the specific area of research [32]. In this case, the stability value is based on the combined estimate of intra- and intergroup variation of gene expression.

## List of abbreviations

OA: Osteoarthritis; RT-PCR: Reverse transcription polymerase chain reaction; qPCR: Quantitative real-time PCR; Ct: Cycle threshold; M: Expression stability measure; HMBS: Hydroxymethyl-bilane synthase; TBP: TATA box binding protein; B2M: Beta-2-microglobulin; ACTB: Beta actin; HPRT1: Hypoxanthine phosphoribosyltransferase 1; RPL13A: Ribosomal protein L13a; SDHA: Succinate dehydrogenase complex; UBC: Ubiquitin C; 18S: Ribosomal protein S18; GAPDH: Glyceraldehyde-3-phosphate dehydrogenase.

## Authors' contributions

MP-S participated in the design of the study, performed the experiments, collaborated in the analysis and interpretation of data and wrote the manuscript. MC collaborated in the analysis and interpretation of data. JJG-R provided critical revision of the manuscript. AG was the principal investigator of the research, conceived and coordinated the study and co-wrote the manuscript. All authors read and approved the final manuscript.

## References

[B1] Woolf AD, Pfleger B (2003). Burden of major musculoskeletal conditions. Bull World Health Organ.

[B2] Felson DT (2004). Risk factors for osteoarthritis: understanding joint vulnerability. Clin Orthop Relat Res.

[B3] Cimmino MA, Parodi M (2005). Risk factors for osteoarthritis. Semin Arthritis Rheum.

[B4] Lapadula G, Iannone F (2005). Metabolic activity of chondrocytes in human osteoarthritis as a result of cell-extracellular matrix interactions. Semin Arthritis Rheum.

[B5] Aigner T, Fundel K, Saas J, Gebhard PM, Haag J, Weiss T, Zien A, Obermayr F, Zimmer R, Bartnik E (2006). Large-scale gene expression profiling reveals major pathogenetic pathways of cartilage degeneration in osteoarthritis. Arthritis Rheum.

[B6] Smith GN (2006). The role of collagenolytic matrix metalloproteinases in the loss of articular cartilage in osteoarthritis. Front Biosci.

[B7] Poole ARGF, Abramson SB, Moskovitz RWAR, Hochberg MC, Buckwalter JA, Goldberg VM (2007). Etiopathogenesis of osteoarthritis. Osteoarthritis.

[B8] Yagi R, McBurney D, Laverty D, Weiner S, Horton WE (2005). Intrajoint comparisons of gene expression patterns in human osteoarthritis suggest a change in chondrocyte phenotype. J Orthop Res.

[B9] Eid K, Thornhill TS, Glowacki J (2006). Chondrocyte gene expression in osteoarthritis: Correlation with disease severity. J Orthop Res.

[B10] Sato T, Konomi K, Yamasaki S, Aratani S, Tsuchimochi K, Yokouchi M, Masuko-Hongo K, Yagishita N, Nakamura H, Komiya S, Beppu M, Aoki H, Nishioka K, Nakajima T (2006). Comparative analysis of gene expression profiles in intact and damaged regions of human osteoarthritic cartilage. Arthritis Rheum.

[B11] Mallein-Gerin F, Gouttenoire J (2004). RNA extraction from cartilage. Methods Mol Med.

[B12] Price JS, Waters JG, Darrah C, Pennington C, Edwards DR, Donell ST, Clark IM (2002). The role of chondrocyte senescence in osteoarthritis. Aging Cell.

[B13] Wang J, Hu L, Hamilton SR, Coombes KR, Zhang W (2003). RNA amplification strategies for cDNA microarray experiments. Biotechniques.

[B14] Subkhankulova T, Livesey FJ (2006). Comparative evaluation of linear and exponential amplification techniques for expression profiling at the single-cell level. Genome Biol.

[B15] Dell'Accio F, De Bari C, El Tawil NM, Barone F, Mitsiadis TA, O'dowd J, Pitzalis C (2006). Activation of WNT and BMP signaling in adult human articular cartilage following mechanical injury. Arthritis Res Ther.

[B16] Gosset M, Berenbaum F, Levy A, Pigenet A, Thirion S, Saffar JL, Jacques C (2006). Prostaglandin E2 synthesis in cartilage explants under compression: mPGES-1 is a mechanosensitive gene. Arthritis Res Ther.

[B17] Soder S, Roach HI, Oehler S, Bau B, Haag J, Aigner T (2006). MMP-9/gelatinase B is a gene product of human adult articular chondrocytes and increased in osteoarthritic cartilage. Clin Exp Rheumatol.

[B18] Stove J, Gremmes C, Gunther KP, Scharf HP, Schwarz M (2006). Metabolic activity and gene expression of osteoarthritic chondrocytes in correlation with radiological and histological characteristics. Biomed Pharmacother.

[B19] Bustin SA, Nolan T (2004). Pitfalls of quantitative real-time reverse-transcription polymerase chain reaction. J Biomol Tech.

[B20] Wong ML, Medrano JF (2005). Real-time PCR for mRNA quantitation. Biotechniques.

[B21] Huggett J, Dheda K, Bustin S, Zumla A (2005). Real-time RT-PCR normalisation; strategies and considerations. Genes Immun.

[B22] Suzuki T, Higgins PJ, Crawford DR (2000). Control selection for RNA quantitation. Biotechniques.

[B23] Lee PD, Sladek R, Greenwood CM, Hudson TJ (2002). Control genes and variability: absence of ubiquitous reference transcripts in diverse mammalian expression studies. Genome Res.

[B24] Vandesompele J, De Paepe A, Speleman F (2002). Elimination of primer-dimer artifacts and genomic coamplification using a two-step SYBR green I real-time RT-PCR. Anal Biochem.

[B25] Dheda K, Huggett JF, Bustin SA, Johnson MA, Rook G, Zumla A (2004). Validation of housekeeping genes for normalizing RNA expression in real-time PCR. Biotechniques.

[B26] Toegel S, Huang W, Piana C, Unger F, Wirth M, Goldring M, Gabor F, Viernstein H (2007). Selection of reliable reference genes for qPCR studies on chondroprotective action. BMC Molecular Biology.

[B27] Glare EM, Divjak M, Bailey MJ, Walters EH (2002). beta-Actin and GAPDH housekeeping gene expression in asthmatic airways is variable and not suitable for normalising mRNA levels. Thorax.

[B28] Ohl F, Jung M, Xu C, Stephan C, Rabien A, Burkhardt M, Nitsche A, Kristiansen G, Loening SA, Radonic A, Jung K (2005). Gene expression studies in prostate cancer tissue: which reference gene should be selected for normalization?. J Mol Med.

[B29] Laidlaw AM, Copeland B, Ross CM, Hardingham JE (2006). Extent of over-expression of hepatocyte growth factor receptor in colorectal tumours is dependent on the choice of normaliser. Biochem Biophys Res Commun.

[B30] Vandesompele J, De Preter K, Pattyn F, Poppe B, Van Roy N, De Paepe A, Speleman F (2002). Accurate normalization of real-time quantitative RT-PCR data by geometric averaging of multiple internal control genes. Genome Biol.

[B31] Dheda K, Huggett JF, Chang JS, Kim LU, Bustin SA, Johnson MA, Rook GA, Zumla A (2005). The implications of using an inappropriate reference gene for real-time reverse transcription PCR data normalization. Anal Biochem.

[B32] Andersen CL, Jensen JL, Orntoft TF (2004). Normalization of real-time quantitative reverse transcription-PCR data: a model-based variance estimation approach to identify genes suited for normalization, applied to bladder and colon cancer data sets. Cancer Res.

[B33] Ramakers C, Ruijter JM, Deprez RH, Moorman AF (2003). Assumption-free analysis of quantitative real-time polymerase chain reaction (PCR) data. Neurosci Lett.

[B34] Pfaffl MW, Tichopad A, Prgomet C, Neuvians TP (2004). Determination of stable housekeeping genes, differentially regulated target genes and sample integrity: BestKeeper – Excel-based tool using pair-wise correlations. Biotechnol Lett.

[B35] Muehleman C, Bareither D, Huch K, Cole AA, Kuettner KE (1997). Prevalence of degenerative morphological changes in the joints of the lower extremity. Osteoarthritis Cartilage.

[B36] Rozen S, Skaletsky H, Krawetz S, Misener S (2000). Primer3 on the WWW for general users and for biologist programmers. Bioinformatics Methods and Protocols: Methods in Molecular Biology.

